# Deoxynivalenol, zearalenone, and *Fusarium graminearum* contamination of cereal straw; field distribution; and sampling of big bales

**DOI:** 10.1007/s12550-015-0220-z

**Published:** 2015-02-11

**Authors:** P. Häggblom, E. Nordkvist

**Affiliations:** Department of Chemistry, Environment and Feed Hygiene, National Veterinary Institute, SE-751 89 Uppsala, Sweden

**Keywords:** Mycotoxin, Straw, Cereal, Sampling, *Fusarium*, DON, ZEN

## Abstract

Sampling of straw bales from wheat, barley, and oats was carried out after harvest showing large variations in deoxynivalenol (DON) and zearalenone (ZEN) levels. In the wheat field, DON was detected in all straw samples with an average DON concentration of 976 μg/kg and a median of 525 μg/kg, while in four bales, the concentrations were above 3000 μg/kg. For ZEN, the concentrations were more uniform with an average concentration of 11 μg/kg. The barley straw bales were all positive for DON with an average concentration of 449 μg/kg and three bales above 800 μg/kg. In oat straw, the average DON concentration was 6719 μg/kg with the lowest concentration at 2614 μg/kg and eight samples above 8000 μg/kg. ZEN contamination was detected in all bales with an average concentration of 53 μg/kg with the highest concentration at 219 μg/kg. Oat bales from another field showed an average concentration of 16,382 μg/kg. ZEN concentrations in the oat bales were on average 153 μg/kg with a maximum at 284 μg/kg. Levels of *Fusarium graminearum* DNA were higher in oat straw (max 6444 pg DNA/mg straw) compared to straw from wheat or barley. The significance of mycotoxin exposure from straw should not be neglected particularly in years when high levels of DON and ZEN are also detected in the feed grain. With a limited number of samples preferably using a sampling probe, it is possible to distinguish lots of straw that should not be used as bedding material for pigs.

## Introduction

Fusarium head blight (FHB) is a recurrent disease of wheat, barley, and other small grains across the world, also including northern Europe, mainly caused by the fungal plant pathogens *Fusarium graminearum* and *Fusarium culmorum* (Bottalico and Perrone 2002). FHB results not only in the premature bleaching of the spikes giving rise to white or pink kernels and lowered grain yields but also in the accumulation of mycotoxins (van der Fels-Klerx et al. 2012). *F. graminearum* and *F. culmorum* are also the causal species for the formation of deoxynivalenol (DON) and its acetylated derivatives, as well as other trichothecenes also including the estrogenic mycotoxin zearalenone (ZEN) (Rodrigues and Naehrer 2012; Tiemann and Dänicke 2007).

The FHB disease cycle starts with the germination of overwintering chlamydospores or mycelia in soil or crop residues, giving rise to the primary inoculum in the spring. The ascospores and/or conidia are then released from the perithecia and are spread by wind or splashing water. When fungal spores land on developing spikes at the time of flowering particularly during moist and warm weather conditions, spore germination and infection of the plant may occur. The considered monocyclic nature of FHB is thought to limit the infection to the primary inoculum released during spring (Wegulo 2012).

However, despite substantial knowledge about the pathogenicity of this plant disease, the present intervention strategies seem to have limited effects on disease mitigation and subsequent accumulation of mycotoxins (van der Fels-Klerx et al. 2012).

Infections by *Fusarium* sp. and DON accumulation in cereals were reported in spindles, glumes, stems, and leaves of the plant, raising the question of systemic fungal growth following FHB infection being responsible for the mycotoxin accumulation (Brinkmeyer et al. 2006; Cowger and Arellano 2013). Several field and experimental studies have shown a positive correlation between DON levels in wheat kernels and the amount of *F. graminearum* DNA (Wegulo 2012). Other studies have shown no colonization of *F. graminearum* or *F. culmorum* in wheat heads despite high DON levels following inoculation of seeds or crowns. Recently, Moretti et al. (2014) in growth chamber experiments showed that *F. graminearum*, inoculated in soil or seeds, can grow systemically in the plant with the exception of kernels and heads. High levels of DON and *F. graminearum* DNA were found in crowns, stems, and straw in contrast to low levels of DON and no fungal DNA in the heads and kernels. Ludewig et al. (2005), following ear infection of spring wheat with *F. graminearum*, showed the DON content of kernels to be less than detected in rachis and straw.

Another factor which may influence the distribution of DON in plant tissues is the water solubility of DON. Several studies have demonstrated translocation of the toxin in the sieve tubes via the xylem or phloem (Kang and Buchenauer 1999; Snijders 2004).

In the last years, different *Fusarium* species infected common cereals particularly in the western part of Sweden and resulted in serious challenges for the cereal and pig industry because of reduced yield and mycotoxin accumulation (Fredlund et al. 2013; Lindblad et al. 2013). DON-producing mold species such as *F. graminearum* and *F. culmorum* were shown to be present, and DON was frequently reported in oats and other cereals.

In a recent survey, we sampled cereals and straw at Swedish pig farms and analyzed for DON, ZEN, T-2, and HT-2 toxin (Nordkvist and Haggblom 2014). The data revealed that DON was almost ubiquitous with 89 % of the samples being contaminated, while ZEN was detected in 54 % of the samples where oats were the cereal grain most frequently contaminated. Higher DON and ZEN concentrations were detected in straw compared to grain harvested in 2011 and 2012. The results clearly indicated straw to be a significant source of DON and ZEN in addition to cereals, however, with large variations between farms.

Trichothecenes have a multitude of effects on eukaryotic cells where the most important seems to be inhibition of protein synthesis (Pinton et al. 2012). Also, DON was shown to inhibit the absorption of nutrients by human epithelial cells “in vitro” (Maresca et al. 2002). Clinical symptoms of trichothecene exposure in animals include feed refusal and weight loss, hemorrhage, emesis, and necrosis of different tissues (Mostrom and Raisbeck 2007). Pigs seem to be the most sensitive animal species to both DON and ZEN exposure from the feed (D’Mello et al. 1999; Dänicke et al. 2004).

In 20–30-kg pigs, the intake of wheat straw was on average 13 % of the diet (van Barneveld 2003), while it was estimated that gestating sows, which are fed restrictively, most likely consume larger amounts. In a recent study, the bioavailability of DON from wheat straw and chaff was investigated in pigs (Rohweder et al. 2013). By measuring the serum concentration of DON, the results clearly indicated that the bioavailability was not affected significantly by feeding straw or a grain matrix.

Clearly, the intake of DON and ZEN from straw may significantly contribute to the mycotoxin exposure in pigs and there will be an increased risk of an exposure exceeding acceptable levels when both the grain and straw are contaminated.

The complexity of *Fusarium* mycotoxin exposure in man and animals is further complicated by the fact that plants are able to modify the mycotoxins into masked mycotoxins, i.e., not extracted by conventional extraction solvents used in the analyses (Berthiller et al. 2005). The possible hydrolysis of masked mycotoxins, which may be present in high amounts during mammalian digestion, raises concern that the parent toxin may be released and absorbed in the intestines and thus contributes to the exposure. In a recent report, a nearly complete hydrolysis of deoxynivalenol-3-β-d-glucoside in the intestinal tract of pigs was observed (Nagl et al. 2014).

For animal welfare reasons, straw is commonly used as bedding material in animal production and access to straw in the pig production is laid down in the Swedish animal welfare regulation (SJVFS 2010:15) where attention is being paid to the amount as well as the hygienic quality of the bedding material.

Because of the documented DON and ZEN contamination of straw, there is a need for practical sampling methods that can be used at pig farms in order to select straw which present no mycotoxin harm to the animals. The fact that sampling is generally conceived as a step of the analytical chain which gives the largest contribution to the measurement uncertainty (Reiter et al. 2011) further emphasizes the development of suitable sampling methods. The sampling uncertainty may be even more pronounced for forages and straw than for grains because of irregular shape, density, and the different anatomical fractions of the plant as opposed to grain being more uniform.

In sampling of forages, two strategies to obtain a large number of incremental samples were reported, namely manual grab sampling (Grimsbo Jewett et al. 2001) and core sampling by drilling into the bales (Schaeffer et al. 2000). In the present study, core sampling was applied to big bales of cereal straw directly after harvest.

The aims of the present study were to (a) study the occurrence of DON and ZEN and the presence of *F. graminearum* and *F. culmorum* in straw bales from wheat, barley, and oats at different pig farms and (b) develop a practical sampling method that could be used at the farm to prevent the use of bedding material with elevated mycotoxin levels that may affect pig health.

## Materials and methods

The 2013 growing season was characterized by less rainfall and higher temperatures compared to both 2011 and 2012 which coincided with a smaller number of reports indicating elevated DON levels from the pre-harvest surveillance in cereals organized by the Swedish grain industry. For that reason, the identification of possible fields suitable for the straw sampling study was based on previous surveillance data for DON from 2011 to 2012.

Suitable fields were localized by collecting 30 to 40 heads by hand from 17 fields in July or August 1 to 2 weeks prior to harvest, respectively. Samples of wheat (spring and winter varieties), triticale, barley, and oats from the southern (county of Skåne) and western (county of Värmland) regions of Sweden were collected. The grains were recovered by manual threshing and dried at room temperature, ground in a shearing mill (Laboratory Mill 3610, Perten Instruments, Sweden), and analyzed for DON following the procedure by the supplier of the lateral flow device Rida®Quick DON (R-Biopharm AG, Darmstadt) within 2 to 3 days.

Fields where DON was detected in kernels prior to harvest in the county of Värmland were selected for the straw sampling. The fields were harvested by a combine harvester, and straw was subsequently pressed in round or square big bales within 2–3 days according to the normal practice of the farmer. Sampling was carried out in late August to mid-September of bales (approximately 150–350 kg/bale) from fields of winter wheat (45 round bales), barley (27 square bales), and oats (18 round bales) within 2 days after baling, respectively. The round bales were 1.2 m wide with a diameter of 1.2–1.4 m, and the square bales were 0.8 × 0.9 × 2.5 m. In addition, 7 round bales of oat straw, from one field in the same region, were sampled inside a shelter after 2 weeks of storage.

The straw samples were collected using a “hay probe bale sampler” sampling probe, 18-mm diameter × 550-mm length (Best Harvest, Largo, FL, USA), powered by an 18-V cordless electric drill. The sampling of round bales was carried out by drilling twice radially 550 mm into the bale, and the straw was mixed to 15- to 30-g dry matter samples. Accordingly, square bales were sampled by drilling once from each short side of the bale. The water content of the samples was estimated after drying at 60 °C overnight (16 h) in a ventilated drying cupboard. All samples were ground on a hammer mill to pass a 1-mm screen before analysis of mycotoxins. The ground samples were stored at room temperature prior to mycotoxin and DNA analysis. The analyses of DON and ZEN were carried out by ELISA (Ridascreen® DON and zearalenone, respectively, R-Biopharm AG, Darmstadt). Limits of detection (LOD) and limits of quantification (LOQ) were 37 and 110 μg/kg for DON and 3.5 and 10.5 μg/kg for ZEN, respectively. Mycotoxin concentrations exceeding the range of the calibration curve (DON 500 μg/kg, ZEN 280 μg/kg) were diluted accordingly.

A subset of seven samples of wheat straw, five samples of barley straw, and eight samples of oat straw were selected to cover the range of DON and ZEN concentrations and were subject to specific DNA analysis for quantification of *F. graminearum* and *F. culmorum.* Before analyses, the samples were further homogenized by pestle and mortar using liquid nitrogen. DNA was extracted and quantified in 0.2 g straw, in parallel as previously described, using the TaqMan® Exogenous Internal Positive Control (Applied Biosystems, CA, USA) (Fredlund et al. 2008; Fredlund et al. 2013). The internal amplification control was amplified to the same level as the negative control, showing that the DNA extract did not contain inhibitory substances influencing the quantification. The lowest DNA concentration with linear amplification was 9 pg/mg straw for *F. graminearum* and 15 pg/mg straw for *F. culmorum*.

For regression analysis of the DNA data versus the contents of DON and ZEN, respectively, all data was log (10)-transformed. The three ZEN concentrations below the quantification limit were treated as upper level, i.e., 3.5 μg/kg.

The different data sets were evaluated by descriptive statistics (mean, median, min, and max values); distribution was studied graphically by the use of histograms. The relationship between contents of DON and DNA from *F. graminearum* was modelled by linear regression after log (10) transformation. A study of sampling frequency was made on data from the wheat field (about 8 ha). Average estimates of DON content were calculated by random sampling of subsets of 2 to 45 (all) bales from the data set. The procedure was repeated 20 times; mean values and standard errors of the mean (SEM) were calculated and evaluated graphically. All calculations were made with MS Excel.

## Results

Results from the screening study revealed DON concentrations of kernels below the detection limit of the test kit <500 μg/kg in the county of Skåne and 600–1800 μg/kg in wheat, oats, and barley from the county of Värmland. The latter region was therefore selected for further sampling studies.

**Fig. 1:**
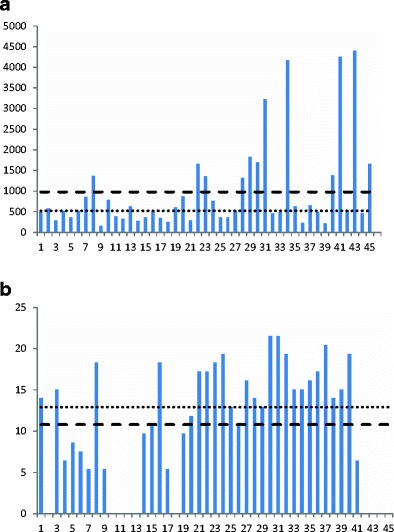
**a** Deoxynivalenol (DON) content (μg/kg) in individual straw bales from one field (8 ha) of winter wheat. Average content is shown as a *dashed line* and median value is represented by a *dotted line*. **b** Zearalenone (ZEN) content (μg/kg) in individual straw bales from one field (8 ha) of winter wheat. Average content is shown as a *dashed line* and the median value is represented by a *dotted line*

The water content of the collected wheat straw samples was between 37 and 64 %; DON was detected in all samples (Fig. 1a) with an average DON concentration of 976 μg/kg and a median of 525 μg/kg, while in four bales, the concentrations were above 3000 μg/kg. For ZEN, eight samples were below the detection limit; however, the concentrations were more uniform compared to corresponding DON data with an average concentration of 11 μg/kg and a median of 13 μg/kg (Fig. 1b).

**Fig. 2:**
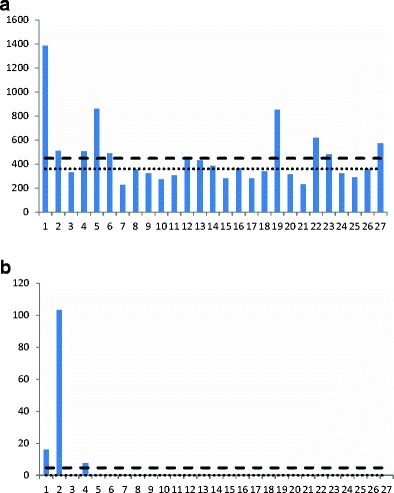
**a** Deoxynivalenol (DON) content (μg/kg) in individual straw bales from one field (8 ha) of barley. Average content is shown as a *dashed line* and the median value is represented by a *dotted line*. **b** Zearalenone (ZEN) content (μg/kg) in individual straw bales from one field (8 ha) of barley. Average content is shown as a *dashed line* and the median value is represented by a *dotted line*

The samples from the barley straw bales contained 18–33 % water and were all positive for DON with an average concentration of 449 μg/kg, and three bales contained levels above 800 μg/kg (Fig. 2a). ZEN was detected in three bales with one sample above 100 μg/kg (Fig. 2b).

**Fig. 3:**
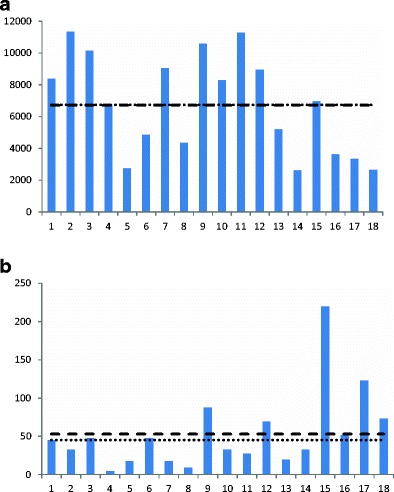
**a** Deoxynivalenol (DON) content (μg/kg) in individual straw bales from one field (5 ha) of oats. Average content is shown as a *dashed line* and the median value is represented by a *dotted line*. **b** Zearalenone (ZEN) content (μg/kg) in individual straw bales from one field (5 ha) of oats. Average content is shown as a *dashed line* and the median value is represented by a *dotted line*

**Fig. 4:**
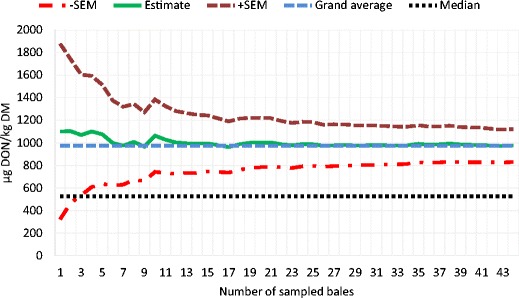
Sampling frequency related to number of sampled bales. Population grand average and median are plotted at respective DON levels (see Fig. 1a)

In the oat straw samples, the water content was between 17 and 23 % and the average DON concentration was 6719 μg/kg (median 6841 μg/kg), with the lowest concentration at 2614 μg/kg and eight samples above 8000 μg/kg (Fig. 3a). ZEN contamination was detected in all bales with an average concentration of 53 μg/kg with the highest concentration at 219 μg/kg (Fig. 3b). In the stored oat bales, the water content was 13–14 % and 5 out of 7 bales were above 15,000 μg DON/kg with an average concentration of 16,382 μg/kg (data not shown). ZEN concentrations in the oat bales were on average 153 μg/kg with a maximum of 284 μg/kg and a minimum at 69 μg/kg (data not shown). In Fig. 4, sampling frequency is related to sampling uncertainty expressed as SEM. It can be seen that sampling only 2 bales at random from the field results in a very high sampling uncertainty. Sampling 5 bales would reduce this uncertainty with about 50 %.

**Fig. 5:**
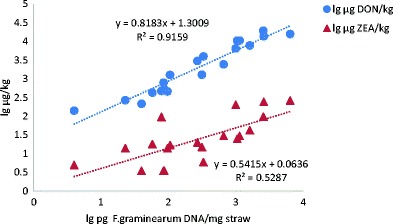
Regression of DON (*filled circles*) and ZEN levels (*filled triangles*) against levels of DNA from *F. graminearum* in straw from winter wheat, barley, and oats (*n* = 20)

*F. graminearum* DNA was present at levels above LOQ in all investigated samples of wheat straw except one, while *F. culmorum* DNA was detected at levels above LOQ in two samples. Levels of *F. graminearum* DNA were higher in oat straw (max 6444 pg DNA/mg straw) compared to wheat and barley. The analyses of DON/ZEN levels and *F. graminearum* DNA levels showed that the mycotoxin levels were significantly correlated with *F. graminearum* DNA (Fig. 5).

## Discussion

The results of the present field study support previous information that DON and ZEN may be present in straw (Nordkvist and Haggblom 2014; Brinkmeyer et al. 2006; Dänicke et al. 2006) even at high levels despite good agricultural practice during the growing and harvest seasons. Interestingly, none of the pig farmers taking part in the study had made observations of FHB—symptoms in their crops and the harvested straw were judged suitable for bedding material.

The water content of the oat straw samples was the lowest followed by barley and winter wheat straw. Because the sampling was carried out only a few days after harvest, except for the stored oat straw, it suggests that the measured straw concentrations of mycotoxins were most likely present at the time of harvest. In experimental studies, Rohweder et al. (2011) observed decreased DON levels and increased ZEN levels when inoculated straw was stored outdoors for longer periods. No kernel contamination in the collected straw samples was observed.

The uneven distribution of mycotoxins in the straw bales from some fields indicated large variations in fungal infections within the field. In winter wheat, four bales contained elevated DON levels (>4000 μg/kg) compared to most bales. Interestingly, those bales were from a slightly lower part of the field possibly with a higher moisture level in the soil.

For ZEN, no obvious correlation with the DON data could be seen, and in about 10 % of the samples, the levels were below LOQ. In barley, DON levels were more uniform in the bales in contrast to ZEN where three samples were above LOQ and one sample above 100 μg/kg.

In contrast to bales from other cereals, the DON and ZEN levels in oats were higher and also the distribution of mycotoxins in oats seemed to be more uniform. Again, the levels of mycotoxins could vary a lot between individual bales with no obvious correlation between DON and ZEN levels, making the sampling rather difficult.

Results from the present field study did not reveal which part of the straw that may harbor mycotoxins at the time of harvest. In experimental studies where winter wheat was inoculated with spores of *F. culmorum*, the mean DON and ZEN concentrations were, however, shown to be significantly higher in glumes and spindles compared to the straw (Brinkmeyer et al. 2006). Further studies should reveal if glumes and spike tissues are the main sources of DON and ZEN in the straw also under field conditions.

Several studies have shown that the amount of DON produced by *F. graminearum* in grain is positively correlated with fungal biomass (Demeke et al. 2010). Other field studies have also shown a positive linear relationship between DON concentrations and the FHB intensity as reviewed in Wegulo (2012).

Results of specific *F. graminearum* DNA showed extensive fungal growth in straw from all tested cereals with the highest concentrations in oats, suggesting a correlation between the amounts of fungal hyphae in the plant and DON levels. When comparing the results in the present study with the data from Fredlund et al. (2013) and Lindblad et al. (2013), considerably higher levels of *F. graminearum*-specific DNA were detected in straw compared to the kernels. In the present study, the range of *F. graminearum* DNA concentrations in oat straw was between 661 and 6444 pg/mg compared to the range in oat kernels of 4–74 and 9–17 pg/mg in 2010 and 2011, respectively. The results clearly suggest a massive fungal colonization in other segments of the plant but the kernel. The results also demonstrate that at the time of harvest, *Fusarium*-infected straw and chaff represent a great risk for spreading *F. graminearum* inoculum to the soil until the next growing season.

Whether the demonstrated straw colonization by *F. graminearum* was the result of the mold being present in the soil or the seeds or infections during flowering is difficult to find out because of diverging reports (Ludewig et al. 2005; Wegulo 2012; Moretti et al. 2014). The farmers participating in the study were all using seeds produced on the farm which may have influenced the infection.

In the present study, four fields were studied to give a rationale for a recommended procedure to be used in practical straw sampling and commercially available equipment designed for sampling of forages was used. Sampling of individual bales on the respective fields made it possible to get information of the in-field variation of the DON and ZEN contamination as the degree of heterogeneity has a great impact on the sampling plan. The use of drilled core sampling has two main advantages, a core sample collected from a cross section of a big bale represents a large proportion of the bale and, secondly, the sample is cut into short pieces that allows for immediate grinding without further preparation of the sample. In the review by Miraglia et al. (2005), it was pointed out that following the sampling variance, the “sample preparation variance” (resulting from subsampling and grinding of the collected (aggregate) sample) was the most significant contributor to the total analytical variation. Thus, grinding the total sampled material will reduce the analytical variation.

In the wheat field, 45 bales were sampled and the distribution was very heterogeneous, as illustrated in Fig. 1a. Half of the bales had a DON content less than 525 μg/kg, while the average content (976 μg/kg) was strongly influenced by four highly contaminated bales. Miraglia et al. (2005) concluded that sampling variance “increases with lowering of the toxin concentration” which is in agreement with the findings in the present work where mean DON level was lower in wheat and barley straw compared to oat straw. For wheat and barley straw, the mean and median values of DON differed greatly with the mean greater than median, reflecting a strong deviation from the normal distribution. It also implies that the average may be of limited use for describing the degree of contamination of individual bales and thus for risk assessment of animal exposure to DON. The more highly contaminated oat straw was less heterogeneous, but still the DON content varied fourfold between the lowest and highest concentrations within one field. A calculation of sampling uncertainty was made for the wheat field. In Fig. 4, the estimates of DON content based on different numbers of sampled bales are shown, where it can be seen that, for this field, sampling 5 bales will result in acceptable sampling uncertainty. However, an estimated mean value of about 1000 DON μg/kg does not imply the absence of highly contaminated individual bales.

Also, the ZEN content of straw varied a lot even if mean and median values were close within the 45 bales of wheat; the distribution was far from normal with <LOQ (3.5 μg/kg) as mode. In the barley field, the contamination was found to be the lowest where the toxin was distributed in three out of 27 bales, giving an average contamination of 5 μg/kg while the median remained at <LOQ.

Additional exposure to DON and ZEN from straw, when used as bedding material, cannot be excluded in pig production particularly as the bioavailability of DON for pigs was shown to be similar to the availability from kernels (Rohweder et al. 2013). The significance of mycotoxin exposure from straw at pig farms with cereal production should for those reasons not be neglected particularly in years when high levels of DON and ZEN are also detected in the feed grain. The present study has demonstrated that estimating the concentrations of DON and ZEN in straw requires a careful sampling plan. However, with a limited number of samples, preferably using a sampling probe, it is possible to distinguish lots of straw that should not be used as bedding material for pigs.
